# Juvenile Idiopathic Arthritis Associated Uveitis

**DOI:** 10.3390/children8080646

**Published:** 2021-07-27

**Authors:** Emil Carlsson, Michael W. Beresford, Athimalaipet V. Ramanan, Andrew D. Dick, Christian M. Hedrich

**Affiliations:** 1 Department of Women’s and Children’s Health, Institute of Life Course and Medical Sciences, University of Liverpool, Liverpool L14 5AB, UK; m.w.beresford@liverpool.ac.uk; 2 Department of Rheumatology, Alder Hey Children’s NHS Foundation Trust Hospital, Liverpool L14 5AB, UK; 3 National Institute for Health Research Alder Hey Clinical Research Facility, Alder Hey Children’s NHS Foundation Trust Hospital, Liverpool L14 5AB, UK; 4 Bristol Royal Hospital for Children & Translational Health Sciences, University of Bristol, Bristol BS2 8DZ, UK; avramanan@hotmail.com; 5 Translational Health Sciences, University of Bristol, Bristol BS2 8DZ, UK; a.dick@ucl.ac.uk; 6 UCL Institute of Ophthalmology, London EC1V 9EL, UK; 7 NIHR Biomedical Research Centre, Moorfields Eye Hospital, London EC1V 2PD, UK

**Keywords:** juvenile idiopathic arthritis, uveitis, biomarkers, risk factors

## Abstract

Juvenile idiopathic arthritis (JIA) is the most common childhood rheumatic disease. The development of associated uveitis represents a significant risk for serious complications, including permanent loss of vision. Initiation of early treatment is important for controlling JIA-uveitis, but the disease can appear asymptomatically, making frequent screening procedures necessary for patients at risk. As our understanding of pathogenic drivers is currently incomplete, it is difficult to assess which JIA patients are at risk of developing uveitis. Identification of specific risk factors for JIA-associated uveitis is an important field of research, and in this review, we highlight the genomic, transcriptomic, and proteomic factors identified as potential uveitis risk factors in JIA, and discuss therapeutic strategies.

## 1. Background

**Figure 1:**
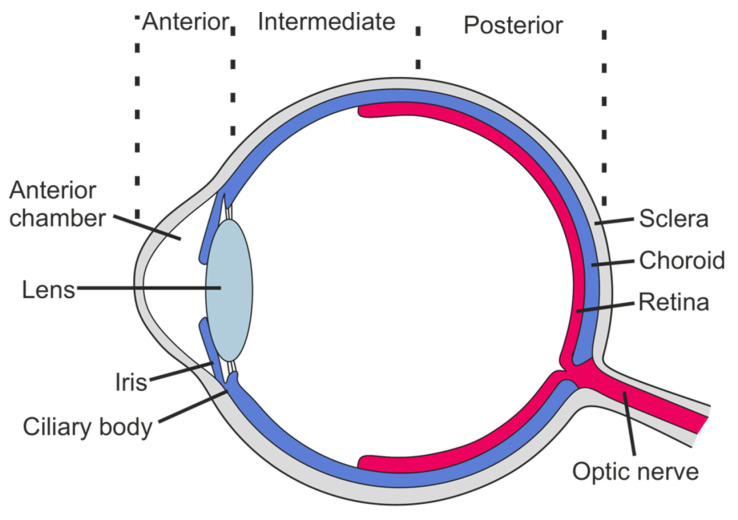
Anatomy of the eye. Cross-section of the human eye with major anatomical features highlighted.

Juvenile idiopathic arthritis (JIA), the most common chronic rheumatic disease in children, has seven sub-forms with variable clinical presentation, disease course, and associated outcomes [1]. Inflammation of the uvea, the pigmented middle layer of the eye comprised of the iris, ciliary body, and choroid, is the most frequent extra-articular manifestation of JIA. Uveitis is classified based on the affected ocular compartment (anterior, intermediate, posterior, or panuveitis) (Figure 1) and the temporal pattern of inflammation (acute, subacute, chronic, or recurrent), with the chronic anterior form being predominant in children with JIA-associated uveitis. While, in most cases, JIA precedes the development of uveitis, a minority of patients develop uveitis before the onset of JIA. Though frequently asymptomatic at onset, JIA-associated uveitis can lead to severe complications, including permanent loss of vision, especially if left untreated [2]. This highlights the urgent need for early detection and initiation of treatment. While the risk of developing uveitis varies between sub-forms of JIA, early diagnosis and treatment are complicated by the current lack of predictive markers for the development of uveitis in individual JIA patients.

## 2. Epidemiology

The prevalence of JIA in developed countries is estimated to range between 16–150 per 100,000 [1,3]. The proportion of JIA patients developing uveitis is up to 30% of individuals positive for antinuclear antibodies (ANA) [4], and 10–20% across all sub-forms of JIA [2,5,6,7,8,9,10,11,12,13,14] (Table 1). While most studies have been performed in primarily White Caucasian cohorts in Western countries, the prevalence of JIA-associated uveitis appears to differ significantly between geographical regions. The highest prevalence is reported in Northern (19.1%) and Southern Europe (18.8%), the lowest in and Latin America (6.4%), Africa, the Middle East (5.9%), and Southeast Asia (5.0%) [15]. A recent study from Japan reported a uveitis prevalence of 6.1% among JIA patients [16].

## 3. Pathophysiology and Systemic Risk Factors

Despite the largely unknown etiology of JIA-associated uveitis [21], several risk factors have been suggested based on demographic and clinical features. These include gender, age at disease onset, and sub-type of JIA+ANA positivity [22]. A young age significantly reduced visual acuity (<0.3) at diagnosis, low intraocular pressure, and sequence of disease events (uveitis before JIA onset) [23,24], and sub-form of JIA also define disease outcomes. Presenting evidence of ongoing disease such as cataracts, synechiae, increased intraocular pressure are characteristics of poor outcomes or ocular damage.

### 3.1. Demographic Risk Factors

Girls, as compared to boys, more frequently develop JIA-associated uveitis. However, as this is in line with the proportion of patients with JIA, it should not be considered an independent risk factor [2,10,14,19,25,26]. Furthermore, age at disease onset is linked to risk. In patients diagnosed with JIA at an early age (≤6 years old), the risk of developing uveitis is notably increased [11,16,27,28], and patients with oligoarticular (as compared to polyarticular) arthritis more frequently develop uveitis [11,19,25,29,30,31,32,33]. As the highest risk for the development of uveitis (approximately 30%) exists in the group of ANA positive oligoarticular JIA patients, approximately 80% of who are girls, all the aforementioned factors may be linked.

Ethnicity may affect the risk of developing JIA-associated uveitis. African American JIA patients develop uveitis less frequently when compared to non-Hispanic White Caucasian patients [5]. However, other studies in multi-ethnic cohorts have not found significant differences in relation to uveitis risk between ethnicities [19].

Associations with female sex, young age, and ethnicity suggest genetic factors as underlying or cause. As uveitis-associated genetic variants are manifold and complex, they will be discussed specifically below. 

### 3.2. Immune Cells and Serum Proteins

The presence of anti-ocular protein autoantibodies has been reported in sera of patients with JIA-associated uveitis [34,35,36,37]. Notably, ANA can also be present in JIA patients without uveitis or healthy individuals, but more commonly occur in uveitis patients, particularly when directed against iris proteins. While it is unknown whether antibody positivity is a cause or result of ocular damage, it may certainly contribute to prolonged local inflammation. A correlation between the presence of serum antibodies binding iris and ciliary body proteins and ocular complications in JIA-associated uveitis has been described [37], which may be used as a future risk stratification tool.

In a small study in 18 children with JIA-associated uveitis, utilizing immunoflow to quantify T-cell surface markers and intracellular cytokines, an increase in the ratio between type 1 T helper cells and type 2 T helper cells (Th1/Th2 ratio) was seen in patients compared with healthy controls [38]. Mean levels were elevated in both JIA-associated and idiopathic anterior uveitis patients, although differences did not reach a significance level. Th1 polarization is believed to result in a predisposition to the development of autoimmune/inflammatory disease and animal models of uveitis have suggested that uveitis is predominantly a Th1/Th17-mediated condition [39,40]. While the study did not detect significant differences in Th17 producing cells between the inflammatory patient groups, Th17 cells across inflammatory disorders were elevated as compared to healthy controls both regarding the quantity of CD4^+^/IL17^+^ cells and mean fluorescence intensity of IL-17 protein.

In a study that analyzed molecular phenotypes of circulating immune cells in patients with JIA-associated uveitis compared to healthy controls or patients with uveitis associated with adult-onset axial spondyloarthritis (SpA), JIA-uveitis patients showed significantly increased numbers of CD56^+^ monocytes and CCR7^+^ dendritic cells (DCs) [41]. While none of the uveitis groups exhibited significant differences in the composition of main monocyte populations (classical, intermediate, or non-classical), JIA patients had increased CD56^+^ classical monocytes as compared to both healthy controls and SpA patients. Indeed, this monocyte population was reduced for SpA patients, which may be associated with the presence of HLA-B27 positivity (18% vs. 95%). Regarding DCs, in line with previous research showing increased expression of the chemokine receptor CCR7 in rheumatoid arthritis [42], JIA-associated uveitis patients also showed an elevated frequency of CCR7-positive myeloid DCs, which may contribute to their recruitment to tissues affected.

Recently, non-biased proteomic and transcriptomic analyses of iris biopsies, aqueous humor (AqH), and serum from JIA-associated uveitis patients revealed differential expression of 136 genes and 56 proteins as compared to patients with primary open-angle glaucoma (POAG) [43], with multiple B-cell related genes upregulated in the uveitis group. These include CD19, CD20, CD27, CD138, and MZB1. Increased concentrations of B-cell survival factors BAFF, APRIL, and IL-6 in the AqH further underscored the probable role of B-cells in the pathogenesis of JIA-associated uveitis. As neither BAFF nor APRIL was found elevated in serum samples, this effect may not be systemic. 

Calcium-binding S100 proteins are a family of low molecular weight proteins secreted by a wide variety of cells. Elevated levels of S100 proteins have previously been described in multiple systemic inflammatory diseases in both children and adults [44,45]. S100A8/A9 heterodimers and S100A12 are elevated in both serum and AqH in JIA-associated uveitis patients as compared to JIA patients without uveitis [46]. Furthermore, serum levels of both S100A8/A9 and S100A12 correlate with uveitis activity. As elevated AqH levels of S100A8/A9 were also reported in idiopathic anterior uveitis and herpetic anterior uveitis, their presence does not appear to be specific for JIA-associated uveitis. S100A8/A9 and S100A12 serum levels were elevated not only in patients with active joint inflammation but also patients with inactive joint inflammation but active uveitis, indicating that this may be a systemic marker of ongoing immune response rather than a specific disease group. Mechanistically, secreted S100 proteins are capable of activating TLR4 [47] and TLR2 [48] signaling, as well as the receptor for advanced glycation end-products [48], thereby activating the innate immune response and production of pro-inflammatory cytokines and chemokines in phagocytes and monocytes.

### 3.3. Environmental Factors

Lastly, environmental factors may be associated with an increased risk of uveitis in JIA [49]. Vitamin D deficiency is associated with multiple autoimmune/inflammatory diseases [50]. Whether an association between low vitamin D levels and the incidence of JIA exists is not clear [51], but data from a German cohort suggests vitamin D deficiency in JIA patients to be associated with higher disease activity and increased risk for the development of JIA associated uveitis [52]. As sun exposure affects vitamin D levels [53], low vitamin D may also contribute to the aforementioned differences in uveitis prevalence between geographic regions with Northern countries having the highest uveitis rates among JIA patients.

## 4. Genetic Factors

In view of the permanent nature of our genetic makeup, the identification of variants that confer elevated risk for the development of uveitis in JIA is highly significant and may be utilized as predictive markers even before disease onset. Demographic and geographic associations suggest genetic variants to contribute to disease risk and expression. Reports of familial clusters exist [54,55,56,57,58,59], but as these are rare, it remains unclear whether they support a role for genetic influence in JIA-associated uveitis pathology.

### 4.1. Human Leukocyte Antigen (HLA)

In keeping with many other autoimmune/inflammatory diseases, polymorphisms in the human leukocyte antigen (HLA) region have been associated with uveitis in JIA. Genome-wide association studies (GWAS) investigating 522 JIA patients, 192 of which experienced uveitis [60], showed that the presence of a serine amino acid residue at position 11 of the YST motif, comprised of tyrosine (Y), serine (S), and threonine (T), of the HLA–DRβ1 peptide-binding groove associates with increased risk for the development of uveitis in girls. The *HLADRB1* gene encodes for the beta-subunit of HLA-DR, a heterodimeric MHC class II cell surface receptor expressed primarily on antigen-presenting cells. Thus, modifications in the peptide-binding groove may affect MHC class II antigen presentation. Interestingly, as HLA-expression on T-cells is considered a marker of T-cell activation, increased expression of HLA-on T-cells has been linked with multiple autoimmune diseases. For example, the proportion of HLA-positive CD8 T-cells has been shown to reflect disease activity in SLE [61], and in Kawasaki disease, increased T-cell HLA-expression is associated with resistance to administration of intravenous immunoglobulin [62]. Another study in Japanese JIA patients reported polymorphisms in HLA-A and HLA-DRβ1 to be associated with risk of uveitis in a cohort of 106 patients (67 with polyarthritis and 39 with oligoarthritis) compared with 678 healthy controls [63]. 

In enthesitis-related arthritis (ERA) JIA, a type of uveitis characterized by acute onset and conjunctival injection, pain, and photophobia occurs in approximately 10% of patients within 6 months of disease onset [64]. This contrasts with uveitis in other forms of JIA, which is often asymptomatic. ERA JIA is defined as arthritis and enthesitis, or arthritis or enthesitis with at least two of the following: the presence of or a history of sacroiliac joint tenderness and/or inflammatory lumbosacral pain, HLA-B27 positivity, the onset of arthritis in a male over 6 years of age, acute (symptomatic) anterior uveitis, or history of HLA-B27-associated disease [65]. HLA-B27 positive ERA JIA patients are at increased risk compared with HLA-B27 negative [20,66]. Uveitis is also a common feature of adult-onset SpA where the vast majority of patients are HLA-B27 positive [67], underscoring the aforementioned potential role for HLA-B27 in uveitis development.

### 4.2. Non-HLA Genes

Studies investigating non-HLA genes that may confer risk of JIA-associated uveitis delivered some conflicting results. Sequencing of the promoter region of *IL1A* in White Norwegian JIA patients delivered an association of a single nucleotide polymorphism (SNP) that was particularly strong in uveitis patients [68]. However, this was not replicated in other studies in White Caucasian patients in the UK [69,70]. 

Retrospective analysis of sequence variants in six genes (*PTPN22, STAT4, TRAF1/C5 locus, TGFB, TNFAIP3,* and *C12orf30*) associated with JIA or other autoimmune/inflammatory conditions did not reveal association with uveitis across all JIA sub-forms [71]. However, the *TRAF1/C5* AA allele at chr9:120942809 (GRCh38.p12; rs10818488) which is in strong linkage disequilibrium with an SNP at chr9:120927961 (GRCh38.p12; rs3761847) in Caucasian and Asian populations [72], while just missing significance level (*p* = 0.06) in oligoarticular and polyarticular RF-negative JIA, is associated with increased uveitis risk in ANA positive oligoarticular and polyarticular JIA patients. The *TRAF1/C5* variants are located near genes encoding for the complement component 5 and the TNF-receptor associated factor 1, a negative regulator of the TNF pathway, underscoring the role of TNF signaling in JIA associated uveitis [73].

Investigation of 17 non-HLA variants associated with JIA in a multicenter study cohort of patients from the Nordic countries identified a polymorphism in the gene encoding the V-set domain-containing T-cell activation inhibitor-1 (VTCN1), also known as B7-H4, to be associated with an increased risk of uveitis [74]. While the confidence interval of the odds ratio was broad (95% CI: 1.01–12.14), this is an interesting finding, as B7-H4 acts as a negative regulator of the T-cell immune response [75].

## 5. Laboratory Biomarkers

The detection and/or quantification of biological markers associating with the presence of uveitis or disease outcomes, such as proteins or immune cell composition in the blood or other tissue, may allow for risk stratification and informed treatment. Several potential markers have been suggested and evaluated in JIA-associated uveitis, with some already used in clinical practice (Table 2).

### 5.1. Anti-Nuclear Antibodies (ANAs)

The most consistent current predictor of uveitis development in JIA is the presence of ANAs. Several studies independently concluded that ANA-positive JIA patients exhibit an increased risk for the development of uveitis [2,4,10,11,14,16,19,25,26,28,76,77,78]. Antinuclear antibodies bind DNA, RNA, nucleosomes, and/or nuclear proteins, and are implicated in the pathophysiology of several autoimmune/inflammatory conditions and therefore routinely tested in clinical settings. The exact mechanistic link between ANAs and uveitis is not known. However, it is noteworthy that B-cells and fully differentiated plasma cells are characteristic of the inflammatory ocular cellular infiltrate in JIA-associated uveitis [88,89,90,91,92]. This, and the high prevalence of ANA positivity, suggest a role of B-cell dysregulation in disease pathophysiology.

### 5.2. Rheumatoid Factor (RF)

Another commonly tested and observed autoantibody is the rheumatoid factor (RF). Notably, RF positivity associates with a relatively low risk of uveitis development [22]. Thus, RF has previously been discussed as having “protective” effects [5]. However, if uveitis occurs in individuals testing positive for RF, it shows more severe clinical courses [93]. The less favorable ocular outcome in RF-positive JIA patients with uveitis may rather reflect reduced differences in the pathophysiology and associated uveitis risk of different JIA sub-forms, rather than having a protective effect as an autoantibody.

### 5.3. Erythrocyte Sedimentation Rate (ESR)

Accelerated erythrocyte sedimentation rates (ESR) have been reported in JIA-associated uveitis patients [14,79,80,81,82,83,84]. As ESR is routinely measured during standard blood tests in JIA, this could potentially provide valuable and cost-effective insight into the risk of developing uveitis. Elevated ESR typically reflects the increased activity of the immune system, which in the context of autoimmune disease could indicate high levels of inflammation. Specifically, for uveitis, a hyperactive immune system may predispose ocular infiltration of reactive immune cells. While this may suggest that ESR would be a useful tool for evaluating the baseline risk of developing uveitis in JIA at the time of diagnosis, it should be noted that normalization of ESR following successful treatment and resolution of inflammation is typically delayed [94]. This makes it less useful for ongoing disease monitoring in response to treatment. Furthermore, elevated ESR may be linked to a number of unrelated factors, such as plasma albumin concentration and patient red blood cell characteristics present in hematological disorders, which are affected by demographic factors (e.g., age, sex). These factors limit the practical usability of the ESR in JIA-associated uveitis disease monitoring [95]. 

### 5.4. Ocular Proteins

Multiplex analysis of 51 inflammatory mediators in AqH and serum samples from JIA-associated uveitis patients compared with chronic anterior uveitis without arthritis, noninfectious idiopathic uveitis and healthy controls identified reduced IL-29 in AqH as a specific marker for JIA-uveitis [85]. Furthermore, AqH levels of osteoprotegerin (OPG) and latency-associated peptide (LAP) were elevated in comparison with the idiopathic uveitis and healthy control groups. When analyzed for differences in serum samples, IL-29 could not be compared as it was not detected in most samples, while OPG and LAP did not show differences between groups. IL-29 (also known as interferon-λ1) is a proinflammatory type III interferon that tightens the blood-brain barrier in response to West Nile infection [96], and could potentially play a similar role in the maintenance of the immune-privileged status of the eye via the blood-retina barrier function. However, elevated levels of interferon-λ are also seen in the retinae of age-related macular degeneration patients, which there provides a signal for homing of neutrophils into the retina by upregulating neutrophil LCN-2 through the STAT1 pathway [97].

Surface-enhanced laser desorption/ionization (SELDI) time of flight (ToF) mass spectrometry analysis of paired serum and AqH samples in children with uveitis delivered distinct differences in AqH proteins between JIA-associated uveitis and other forms of childhood uveitis and healthy controls [86]. The study identified transthyretin (TTR) as a potential local marker for JIA-associated uveitis, as AqH levels were increased in JIA and silent chronic anterior uveitis, while the protein was not detected in serum. Ocular TTR is abundantly produced and apically secreted by the retinal pigment epithelium in a polarized manner [98]. It has amyloidogenic properties and has been found in drusen [99], the extracellular subretinal deposits frequently observed in age-related macular degeneration.

Because of the relative difficulty and invasiveness of sampling AqH, tear collection has been suggested for protein analysis in the context of uveitis. In a pilot study on three children with JIA-uveitis, elevated levels of proteins associated with inflammatory arthritis (SEMA3G, TIMP1, HEXB, ERN1, and SAA1) was found, in addition to elevated levels of sCD14, S100A8, and SAA1, but reduced levels of S100A9, LAP3, TTR, and MIF [87]. Previously, tear levels of IL-1RA, IL-8, fractalkine, IP-10, VEGF, and TGF-β2 had been shown to be elevated in uveitis [100], although this study encompassed multiple diverse forms of uveitis and not specifically JIA-associated uveitis.

## 6. Treatment

Management of JIA-uveitis encompasses multiple aspects, including screening, joint pediatric rheumatology and ophthalmology clinics, and multidisciplinary care. As evidence-based guidelines are relatively sparse, regimens are largely based on physicians’ experience. Initiatives to provide international expert consensus opinions on diagnosis and treatment of JIA-uveitis, such as those developed by the Single Hub and Access point for pediatric Rheumatology in Europe (SHARE) initiative [101] have been developed to identify best practices in treatment and care. 

Regarding pharmaceutical interventions, first-line treatment strategies for JIA-associated uveitis include topical glucocorticoids, and in the case of synechiae, mydriatic eye drops to prevent an increase in intraocular pressure and cataract development. If topical treatment fails to induce stable remission, systemic treatments are added, which may include corticosteroids, disease-modifying antirheumatic drugs (DMARDs), and biopharmaceutical agents [102,103,104,105]. Regarding the use of corticosteroids, paucity of evidence underpins the most effective route of administration (oral vs. intravenous), dosing, and treatment duration. A recent retrospective study suggested intravenous methylprednisolone pulse treatment to be effective in limiting inflammatory activity in uveitis at diagnosis and during disease flares, even when already treated with second-line agents [106]. However, based on small case numbers, the study was unable to draw reliable conclusions on how many pulses (namely, one versus three or more in monthly intervals) may be necessary to induce stable remission [106]. 

If corticosteroid treatment fails or flares occur after their discontinuation, several DMARDs may be used to achieve or maintain remission. Methotrexate is the standard first-line DMARD used and is generally considered more effective as compared to azathioprine or mycophenolate mofetil [107,108]. Although T-cell inhibition mediated via cyclosporine has been shown to be effective against idiopathic uveitis [109], the drug was found to be of limited efficacy in JIA-associated uveitis [110], suggesting different or additional mechanisms or immune cell involvement for disease progression. 

In patients with failing or with insufficient response to DMARDs, biopharmaceutical drugs will be used, usually targeting TNF and IL-6 pathways [104]. The SYCAMORE trial [111] demonstrated the significant benefit of anti-TNF treatment (adalimumab) in patients failing on methotrexate, and the ADJUVITE trial [112] was in favor of using adalimumab in patients with early-onset, chronic anterior uveitis, which is in most cases associated with JIA, in case of inadequate response to topical therapy and methotrexate. The APTITUDE trial [113] showed mixed results in using anti-IL6 treatment (tocilizumab). Although the endpoint was not met to determine evidence for a phase III trial, one-third of participants had a two-step improvement in uveitis assessment at week 12, and three of four participants had complete resolution of cystoid macular edema. Notably, it should be noted that the APTITUDE trial was focused on patients refractory to anti-TNF treatment. Tocilizumab may therefore provide a therapeutic option in some children with uveitis refractory to anti-TNF drugs, given the absence of other treatment options. Additional and/or future candidates for application in JIA-associated uveitis currently being evaluated include small-molecule Janus kinase (JAK) inhibitors, and biopharmaceutical drugs, such as IL-17 inhibitors, B-cell depletion (e.g., through anti-CD20 antibodies), and T-cell inhibition (e.g., through CTLA-4) [114]. For future trials and basic science studies, ANA positive chronic anterior uveitis should be considered the same disease as JIA-associated uveitis.

A central challenge in the therapeutic management of JIA-associated uveitis concerns surgical management of complications, e.g., cataracts and glaucoma, where adequate immunosuppression is essential with no tolerance for even mildly active ocular inflammation. Possible postoperative (and inflammation-associated) complications include synechiae, cyclitic membrane, postoperative glaucoma, and macular edema, and consequently, the therapeutic approach must also seek to improve outcomes in these patients [115]. Future care will involve close interdisciplinary collaboration between pediatricians and immunologists to improve outcomes.

Currently, a central problem with determining the optimal treatment strategy for JIA-associated uveitis is that predictive biomarkers for treatment response (or non-response) are lacking. While the strong benefits of early detection of uveitis and initiation of treatment are well established, valuable time can be lost due to selecting a sub-optimal drug. Identification of reliable biomarkers will help to inform treatment strategy and should therefore be considered a high priority in ongoing research. These issues are currently being addressed by the MRC-funded UK-wide CLUSTER Consortium (https://www.clusterconsortium.org.uk accessed on 21 June 2021) and other international collaborative initiatives.

## 7. Conclusions and Future Directions

JIA is the most common childhood rheumatic disease. Associated uveitis can result in severe associated complications. The risk of developing uveitis in JIA has to date mainly been evaluated in relation to demographic and clinical factors. Recent research efforts have increasingly employed molecular proteomic and cellular phenotyping tools with the aim of identifying patient-specific markers that modulate the overall risk. As several new therapeutics are considered as options for clinical use, biomarker discovery studies are of critical importance for future ability to determine optimal strategies for personalized medicine. Although an ideal biomarker would be a single factor that is highly specific and sensitive, more realistic outcomes of these studies would be multifactorial panels of biomarkers that combined can inform individual risk of developing uveitis in JIA, as well as treatment response for patients with diagnosed JIA-associated uveitis.

## Figures and Tables

**Table 1:** Juvenile idiopathic arthritis (JIA) and relative risk of uveitis.

Type of JIA	Sex Ratio (F:M)	Age of Uveitis Onset	Uveitis Risk	Type of Uveitis
Systemic arthritis	1:1	N/A	Low	N/A
Oligoarthritis (persistent)	3:1	Early childhood	10–20%	Chronic asymptomatic
Oligoarthritis (extended)	3:1	Early childhood	20–30%	Chronic asymptomatic
Polyarthritis (RF-)	3:1	Early childhood	10–20%	Chronic asymptomatic
Polyarthritis (RF+)	4:1	N/A	Low	N/A
Psoriatic arthritis	2:1	Early childhood or school-age	5–20%	Chronic asymptomatic or acute symptomatic
Enthesitis related arthritis	1:4	School-age	5–20%	Acute symptomatic

Approximate numbers estimated from [5,6,9,10,14,16,17,18,19,20]. RF, rheumatoid factor.

**Table 2:** Potential biomarkers in JIA-associated uveitis.

Risk Factor	Tissue	Status in JIA-Uveitis	Comparator Group	Reference
ANA	Blood	Higher proportion positive	JIA	[2,4,10,11,14,16,19,25,26,28,76,77,78]
ESR	Blood	Increased rate	JIA	[14,79,80,81,82,83,84]
Anti-ocular antibodies	Serum	Higher proportion positive	JIA, idiopathic arthritis and healthy controls	[34,35,36,37]
BAFF	AqH	Increased	POAG	[43]
APRIL	AqH	Increased	POAG	[43]
IL-6	AqH	Increased	POAG	[43]
IL-29	AqH	Reduced	JIA	[85]
OPG	AqH	Increased	Healthy controls and idiopathic arthritis	[85]
LAP	AqH	Increased	Healthy controls and idiopathic arthritis	[85]
TTR	AqH	Increased	Other uveitis and non-inflammatory controls.	[86]
S100A8/A9	AqH/Serum	Increased	JIA	[46]
S100A12	AqH/Serum	Increased	JIA	[46]
SEMA3G	Tears	Increased	Idiopathic uveitis	[87]
TIMP1	Tears	Increased	Idiopathic uveitis	[87]
HEXB	Tears	Increased	Idiopathic uveitis	[87]
ERN1	Tears	Increased	Idiopathic uveitis	[87]
SAA1	Tears	Increased	Idiopathic uveitis	[87]
sCD14	Tears	Increased	Idiopathic uveitis	[87]
S100A8	Tears	Increased	Idiopathic uveitis	[87]
S100A9	Tears	Reduced	Idiopathic uveitis	[87]
LAP3	Tears	Reduced	Idiopathic uveitis	[87]
TTR	Tears	Reduced	Idiopathic uveitis	[87]
MIF	Tears	Reduced	Idiopathic uveitis	[87]

ANA, anti-nuclear antibodies; ESR, erythrocyte sedimentation rate; BAFF, B-cell activating factor; APRIL, a proliferation-inducing ligand; OPG, osteoprotegerin; LAP, latency-associated peptide; TTR, transthyretin; SEMA3G, semaphorin 3G; TIMP1, tissue inhibitor of metalloproteinases 1; HEXB, hexosaminidase subunit beta; ERN1, endoplasmic reticulum to nucleus signaling 1; SAA1, serum amyloid A1; sCD14, soluble CD14; LAP3, latency-associated peptide; MIF, macrophage migration inhibitory factor; AqH, aqueous humor; POAG, primary open-angle glaucoma.

## Search Strategy and Review Criteria

We searched the MEDLINE database via PubMed for relevant published articles in English language published before April 2021. Search terms used were “juvenile idiopathic arthritis” and “uveitis”, or a number of synonyms to these terms, including “juvenile rheumatoid arthritis”, “juvenile chronic arthritis”, “juvenile arthritis”, “jia”, “iritis”, and “iridocyclitis”. The reference lists of selected articles were also reviewed for additional relevant papers. All articles considered relevant were taken into account, with an emphasis for articles published in the last 5 years.
